# Designing the Slide-Ring Polymer Network with both Good Mechanical and Damping Properties via Molecular Dynamics Simulation

**DOI:** 10.3390/polym10090964

**Published:** 2018-09-01

**Authors:** Zhiyu Zhang, Guanyi Hou, Jianxiang Shen, Jun Liu, Yangyang Gao, Xiuying Zhao, Liqun Zhang

**Affiliations:** 1 Key Laboratory of Beijing City on Preparation and Processing of Novel Polymer Materials, Beijing University of Chemical Technology, Beijing 100029, China; zhangzy59908@163.com (Z.Z.); hgy1939916@163.com (G.H.); gaoyy@mail.buct.edu.cn (Y.G.); zhaoxy@mail.buct.edu.cn (X.Z.); 2 Department of Polymer Science and Engineering, Jiaxing University, Jiaxing 314001, China; Jianxiang@gmail.com; 3 Beijing Engineering Research Center of Advanced Elastomers, Beijing University of Chemical Technology, Beijing 100029, China; 4 Engineering Research Center of Elastomer Materials on Energy Conservation and Resources, Beijing University of Chemical Technology, Beijing 100029, China; 5 Beijing Advanced Innovation Center for Soft Matter Science and Engineering, Beijing University of Chemical Technology, Beijing 100029, China; 6 State Key Laboratory of Organic-Inorganic Composites, Beijing University of Chemical Technology, Beijing 100029, China

**Keywords:** slide-ring polymer, dynamic properties, chain sliding

## Abstract

Through coarse-grained molecular dynamics simulation, we have successfully designed the chemically cross-linked (fixed junction) and the slide-ring (SR) systems. Firstly, we examine the dynamic properties such as the mean-square displacement, the bond, and the end-to-end autocorrelation functions as a function of the cross-linking density, consistently pointing out that the SR system exhibits much lower mobility compared with the fixed junction one at the same cross-linking density. This is further validated by a relatively higher glass transition temperature for the SR system compared with that of the fixed junction one. Then, we calculated the effect of the cross-linking density on the stretch-recovery behavior for the SR and fixed junction systems. Although the chain orientation of the SR system is higher than that of the fixed-junction system, the tensile stress is smaller than the latter. We infer that much greater chain sliding can occur during the stretch, because the movable ring structure homogeneously sustains the external force of the SR system, which, therefore, leads to much larger permanent set and higher hysteresis during the recovery process compared with the fixed-junction one. Based on the stretch-recovery behavior for various cross-linking densities, we obtain the change of the hysteresis loss, which is larger for the SR system than that of the fixed junction system. Lastly, we note that the relatively bigger compressive stress for the SR system results from the aggregation of the rigid rings compared with the fixed junction system. In general, compared with the traditionally cross-linked system, a deep molecular-level insight into the slide-ring polymer network is offered and thus is believed to provide some guidance to the design and preparation of the slide-ring polymer network with both good mechanical and damping properties.

## 1. Introduction

It is well known that cross-linked polymeric gels have attracted much attention because of their significant practical applications, such as actuators for optics, vehicles for drug delivery and release, and matrices for electronics and tissue engineering [1,2,3,4,5,6]. The polymeric gels are divided into chemical gels [7,8] and physical gels [9], based on covalent and non-covalent bonds such as hydrogen bonds and the hydrophobic interaction [10]. The properties of physical gels are more likely influenced by the high temperature and the solvent, which determine the instability of the non-covalent bonds [11]. However, the covalent bond of the chemical gels is much more stable. Although the construction of the cross-linked covalent bonds [12] is common for polymeric materials, the fixed junction sites in the cross-linked network exhibit some disadvantages, such as the tendency to form the stress concentration leading to the early fracture [13]. In order to resolve this challenge, Ito [14] finally proposed the slide-ring gels (SR gels) and the slide-ring materials (SRM) with movable junctions.

As for the practical applications of SRM, it is generally accepted that this kind of structure can help to redistribute the external stress. Therefore, those materials can be applied as damping materials and flexible matrices. According to the experimental results from Wang et al. [15], a novel isolation bearing was successfully fabricated by introducing the SRM, exhibiting high damping and mechanical property [16]. Besides, Howell et al. [17] introduced the SR gels to the elastic organic/inorganic composite photonic crystals, which finally exhibited desirable flexibility. In addition, Yang et al. [18] fabricated a kind of dielectric elastomer material with the SRM, and the material exhibited a low elastic modulus and high electromechanical sensitivity. Those applications above have shown good scientific and technological potentials of SRM, attributed to the topological structure in the SR gels.

In the synthesis [19,20], the SR gels are generally made from cross-linked polyrotaxanes (PRs), a kind of complex molecule consisting of several α-cyclodextrin (α-CD) rings, poly(ethylene glycol) (PEG) as backbone chains, and bulky capping ends. Especially, the chemical cross-linking in the SR gels only occurs between the ring structures, which finally enables the connecting pattern being “figure of eight” [21]. The cross-linked structure represents a new kind of cross-linking, which differs from the conventional fixed cross-linked junction. Therefore, the PEG chains are not completely fixed and can move freely in the polymer network. However, they are still restricted by the terminal ends. The SR gels exhibit different properties compared with the commonly fixed junction, because the movable junction shows the unique “pulley effect” [14] in SR gels. Meanwhile, the “figure of eight” interlocked junction can move freely along the backbone, and easily distributes the stress between the cross-linked sites. However, the heterogeneity of the slide-ring network may occur because of the aggregation of the rings under artificial deformation [22]. For the mechanical behavior, SR gels show a J-shaped stress-strain curve. Generally, the SR gels exhibit lower mechanical property than chemical gels during the deformation owing to the sliding motion of the backbone called the “pulley effect”. Meanwhile, the stress-strain behavior during the deformation in fixed junction systems and slide-ring junction systems can be described through the three Gaussian chains model, as well as its modified version [23], respectively, and those models fit the experiment well. Kato et al. [24] also studied the compression deformation behavior and inferred that the aggregation of ring can contribute to the hardening of the compression stress compared with the conventional polymer gels.

As for the other research of slide-ring materials, Kazuki et al. [25] have developed a kind of organic-inorganic hybrid SRM to achieve better thermostability, as well as gas permeability by introducing silicone into SRM; they further explored its visco-elastic relaxation to reflect the sliding dynamics, as well as the mobility of ring structure. In the work by Ito [26], the SR gels may show the sliding transition after the elasticity plateau. The volume phase transitions of SR gels were observed by examining the swelling-shrinking behavior [27]. In addition, the small-angle neutron scattering (SANS) accurately described the deformation mechanism of the SR gels [28]. Besides, some simulation work has also been carried out, such as the Brownian dynamics simulation concentrating on the sliding junction [29] and a derivation describing a molecular shock absorber [30]. These studies, in particular, have stated that the slide-ring junction has a great effect on the mechanical and damping properties.

Even so, not all properties about SRM could be described comprehensively through experiments, such as the motion of rings and the hysteresis behavior. For SRM, an important feature is the “pulley effect” of the entire material. The “pulley effect” in the SRM is not directly demonstrated with a measure related to mobility, and it is difficult to find a direct compared experiment to highlight the effect of rings (CDs in the SRM) structure. A visualized or distinct measurement on the effect of “moveable or sliding junction” is essential for the further research about SRM. In addition, the “pulley effect” is of fundamental importance, particularly for mechanical and damping properties, and the dependence of those properties on the microstructure of SRM remains elusive.

Analogous to experiments, computer simulation techniques provide an attractive approach to probe the structure-property relationships of SRM. In the MD simulation, it is convenient to achieve the visualization [31,32] of the whole system and carry out the quantitative analysis. The focus of such MD simulation studies has been on the dynamics, stretch-recovery, and compression-recovery behavior [33] and visco-elasticity [34], as well as hysteresis loss of all kinds of materials [35]. For instance, the bead-spring model was always used for revealing the visco-elasticity of elastomeric polymer materials via the visualization of the deformation process by Liu et al. [36]. Also, the coarse-gained model is usually used as a precursor to simplify the systems, and then a fully atomistic model is set to further explore the reinforcement in elastomer nanocomposites, as Pavlov et al. [37] reported.

In our works, we try to investigate the effect of the ring structure on the mechanical and damping properties. To compare the SR structure (figure of eight cross-linking) and the traditional cross-linked elastomer, a simple coarse-grained model with prominent cross-linked ring structure is adopted in our work. In addition, we adopt the coarse-grained model for two main reasons: the first aspect is that we can more conveniently and effectively construct the slide-ring polymer system, especially the rings threading on the backbone of the SR system. The second aspect is that it is relatively easy to reach the equilibrated state and perform the following mechanical deformation. These two aspects are relatively more difficult to realize in the united or fully atomistic models. Compared with the atomistic modeling, it is convenient for us to introduce the cross-linking bonds to the systems without introducing extra atoms and regulate the cross-linking density. Meanwhile, by building another simple fixed junction model as a reference, the CG model of SR systems have included the ring structure as the key factor. We hope that it may reveal some universal conclusions on the SR structure about the dynamics and the chain sliding. Motivated by such consideration, in our work, we build a series of fixed junction and slide-ring systems via coarse-grained MD simulation. These two kinds of systems are set to characterize the sliding junction in the SRM, compared with the conventional cross-linking way in the chemical gels [38] or the conventional cross-linked elastomer [39]. The behavior of SR systems under stretching deformation may provide abundant evidence of the “pulley effect”. Given that the cross-linking density of SR systems adds an adjustable parameter for tailoring the mechanical and damping properties, our results could provide guidelines for the optimization and design of such SRM for special uses.

## 2. Simulation and Method

### 2.1. Model and Force Field

**Figure 1:**
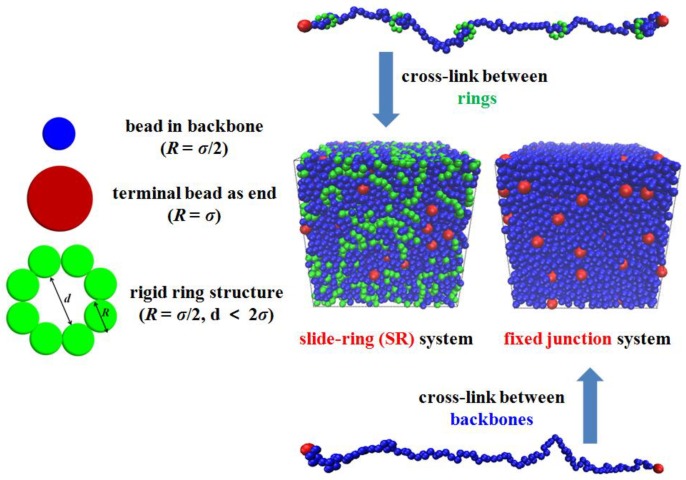
Schematic of the coarse-grained model involved in the simulation. The snapshots show that the SR system consists of the slide-ring chain structure, whose cross-link happens between rings’ structure. Meanwhile the fixed junction system consists of linear chain structure, which represents the chemically cross-linked systems. The blue, red, and green sphere represents the bead in the backbone, end and ring structure, respectively.

In the simulation, we use the coarse-grained (CG) bead-spring model [40] to construct the simplified SR network. It is not our target to consider any specific SR gel or SR polymeric materials but to study this kind of particular topological structure. We define the energy ε, mass m, and diameter σ as the units of the parameters to describe the model, so the expression and calculations are dimensionless. Each SR system includes three kinds of beads, while the fixed junction system has two kinds of beads. The total number of beads in SR systems is 14,000, consisting of 9800 beads in backbone, 4000 beads dividing into 500 rigid rings uniformly threading on all backbones, and 200 beads as the terminal ends of 100 chains. All chains in the systems equally contain 100 beads (including ends). Additionally, each fixed junction system has 10,000 beads and contains the same number of beads in backbone and terminal ends but no ring structure. The beads in the backbone and rings are identical, and their diameter is σ, while the terminal beads are much bigger, and their diameter is 2σ. In the simulation, we set the mass of backbone and ring beads as m, so the mass of terminal beads equals 8m. This difference helps prevent those topological structures from breaking down. The SR system shown in Figure 1 is composed of the single SR structure, while the fixed junction system consists of the same linear structure with terminal ends. The method that was used to build the initial configuration was involved in the supporting information. Then, we cross-link the SR structures by chemically bonding the rings’ structure, rather than bonding the backbones. In our simulation, we realize the cross-linking process as follows: For SR systems, after enough initial equilibrium mentioned above, the permanent cross-linking bonds are imposed in the system, by randomly selecting one pair of beads, which belongs to two different rings. However, the two rings cannot be selected from the same SR structure, which ensures that cross-linking occurs between rings on different SR chains, rather than in a single SR chain. If the distance between the two beads is smaller than 1.12σ, then bonded energy via the harmonic potential is produced between those two randomly chosen ring beads. Similarly, for fixed junction systems, the two beads are randomly selected from different backbones. Also, the distance criteria is 1.12σ [36,41].

To explore the influence of the cross-linking density on the dynamics of the backbone, we get those systems cross-linked with the different numbers of the cross-linked bond, represented by Nc. For all cases in the simulation, the variable Nc is varied as follows: Nc=0 (no cross-link happens), Nc=200, Nc=300, and Nc=400.

To facilitate the molecular dynamics (MD) simulation process, the total force field can be described as follows:

(1) $$E_{\mathrm{total}}=E_{\mathrm{non}-\mathrm{bonding}}\left(r\right)+E_{\mathrm{bond}}\left(r\right)$$

The expanded, truncated, and shifted Lennard-Jones (LJ) interaction is used to model the non-bonding interaction $E_{\mathrm{non}-\mathrm{bonding}}\left(r\right)$ between all kinds of beads in the structure, as follows:

(2) $$U_{ij}\left(r\right)=\{\begin{array}{cc} 4\varepsilon_{ij}\left[{\left(\frac{\sigma }{r-r_{\mathrm{EV}}}\right)}^{12}-{\left(\frac{\sigma }{r-r_{\mathrm{EV}}}\right)}^{6}\right]-U(r_{\mathrm{cutoff}}), & 0<r-r_{\mathrm{EV}}<r_{\mathrm{cutoff}}\\ 0, & r-r_{\mathrm{EV}}\geq r_{\mathrm{cutoff}} \end{array}$$

in which $r_{\mathrm{cutoff}}$ determines the distance ($r-r_{\mathrm{EV}}$) in which the interaction is truncated and shifted so that the energy is zero and r is the distance between two interaction sites (the center of mass). Additionally, we offset the interaction by $r_{\mathrm{EV}}$ for the excluded volume. Here, $U(r_{\mathrm{cutoff}})$ is a constant to maintain the continuity of the interaction. For the backbone–end interaction, $r_{\mathrm{EV}}=\sigma /2$. Moreover, $r_{\mathrm{cutoff}}$ and $\varepsilon_{\mathrm{backbone}-\mathrm{end}}$ are set to $2\times 2^{1/6}\sigma$ and 5.0ε, respectively, because the ends are much bigger compared to the beads in the backbone, so the attraction between ends and backbone is slightly weak. Additionally, it is significant to prevent the ends from aggregating and keep a homogeneous network. For the backbone–ring interaction, $r_{\mathrm{EV}}$ equals zero, and $r_{\mathrm{cutoff}}$ and $\varepsilon_{\mathrm{backbone}-\mathrm{ring}}$ are set to 2 × 2^1/6^*σ* and 1.0ε, respectively, because the sliding activity between backbone and rings is essential, so the attraction is weak. As for the end–end interaction, the end-ring interaction, and the ring-ring interaction, the interaction above is identical, and $r_{\mathrm{cuttoff}}$ and $\varepsilon_{\mathrm{end}-\mathrm{end}}$ are set to $2^{1/6}\sigma$ and 1.0ε. Furthermore, the corresponding $r_{\mathrm{EV}}$ are 1.0σ, 0.5σ, and 0, respectively. All of the parameters describing the non-bonding interaction are presented in Table 1.

The bond stretching energy between the adjacent beads is modeled via the harmonic bond potential:

(3) $$E_{\mathrm{bond}}\left(r\right)=k/2{\left(r-r_{0}\right)}^{2}$$

in which k is the stiffness constant, r is the current bond length, and $r_{0}$ is the equilibrium bond length. To simplify the bond-stretching interaction, only four kinds of bonds are considered in the coarse-grained model. The k and $r_{0}$, which describe the bonds connecting beads in backbones, are set to $100\varepsilon /\sigma^{2}$ and 1.0σ, respectively. In addition, the k and $r_{0}$ in the bonds, which connect the backbone beads and end beads, are set to $180\varepsilon /\sigma^{2}$ and 1.5σ, respectively. Besides, the cross-linking bonds are set to $100\varepsilon /\sigma^{2}$ and 1.0σ in all systems. The mathematical expression of the potentials is well presented in the Table 2; however, the bond stretching energy intra-ring is not considered here, for the rings are modeled as rigid bodies.

### 2.2. Equilibrium Simulation

In the MD simulation process, we use normal pressure and temperature (NPT) ensemble to equilibrate all the systems by employing the Nosé-Hoover temperature thermostat and pressure barostat [42]. Here, we adopt a equilibration process including different temperature ensembles as follows: Firstly, we place all the polymer chains in a very big simulation box, which is relaxed in the NPT ensemble at the fixed temperature $T^{*}=1.0$ and fixed pressure $P^{*}=1.0$. The aim of such a procedure is to compress the systems volume so as to increase the density of polymer beads, around $\rho^{*}=0.85$. Then, we introduce the crosslinks to the systems and fix the temperature of the systems at $T^{*}=2.0$ in the NVT ensemble. In this stage ($T^{*}=2.0$), the equilibrium process at high temperature is performed over a long time (more than $2\times 10^{7}$ timesteps) to ensure each polymer chain has moved at least $2R_{g}$ ($R_{g}$ is the radius of gyration of polymer chain). Finally, the system is further equilibrated under the NPT ensemble with $T^{*}=1.0$ for at least $2\times 10^{7}$ timesteps. The temperature and pressure during the equilibrium is fixed at $T^{*}=1.0$ and $P^{*}=1.0$ for measuring the dynamics. [40]. In our simulation, the averaged number densities of polymer beads in the chemical gel system and SR system are 0.88 and 0.87, respectively. Periodic boundary conditions in three directions are also employed in the simulation. Meanwhile, the velocity-Verlet algorithm is used in the equations of motion to describe the motion of all the beads with the time units $\delta t=0.001t^{*}$, which is reduced by the LJ time (τ). As for the measure of the glass-transition temperature, we change the equilibrium state temperature form $T^{*}=1.0$ to the specific temperature and run for enough time. Then, we plot the volume-temperature curves and use the linear fitting to get the glass-transition temperature.

### 2.3. Non-Equilibrium Simulation

Generally, we obtain the stress-strain curves in the *z* direction for the measure of the mechanical properties. The temperature and pressure for simulating deformation are $T^{*}=1.0$ and $P^{*}=1.0$, respectively. Also, to carry out the uniaxial tension deformation [43] in the simulation, all the systems are under the constant volume state and are deformed by changing the length of the box, $L_{z}(0)$ to $L_{z}(0)a$, in the elongated direction, and to $L_{z}(0)a^{-1/2}$ in the other two directions. The stress $\sigma_{T}$ in the *z* direction is represented by the deviatoric tensor, as follows:

(4) $$\sigma_{T}=\left(1+\mu \right)\left(-p_{zz}+p\right)\approx 3(-p_{zz}+p)/2$$

in which $p=\sum {}_{i}p_{ii}/3$ is the hydrostatic pressure and μ represents the Poisson’s ratio. For the incompressible rubber network, we choose μ=0.5. Meanwhile, $\left(L_{z}(t)-L_{z}(0)\right)\cdot L_{z}{\left(0\right)}^{-1}$ is the strain $\varepsilon_{\mathrm{stretch}}$ at the time t, and the tensile strain rate, ${\dot{\varepsilon }}_{\mathrm{stretch}}$, is defined as follows [36]:

(5) $${\dot{\varepsilon }}_{\mathrm{stretch}}=0.0327/\tau$$

in which $L_{z}(t)$ stands for the length of the box in the elongated *z* direction with the time t increasing, and τ is the unit of the time. Besides, the recovery strain rate is completely identical to the stretch strain rate. We run $1.5\times 10^{5}$ timesteps equally in the stretch and recovery deformation, and the maximum strain in the stretching process is 4.905 [44]. In addition, the compression strain rate is defined as

(6) $${\dot{\varepsilon }}_{\mathrm{compression}}=0.00327/\tau$$

in which the slower compression strain rate is adopted according to the experiment [24]. A sufficient slow compression rate is adopted to characterize the compression behavior, which is clearly slower than the rate in the measurement of the tensile stress-strain behavior in this experiment. Notably, we also run $1.5\times 10^{5}$ timesteps equally for the compression and recovery deformation to maintain the accuracy of the compression stress-strain curves (also 1.5 × 10^5^ time-steps in the stretch deformation). The deformation direction is along the *z* direction, and the maximum strain for the compression is 0.4905 [41]. All the symbols above are identical to those in stretch-recovery deformation. Notably, the snapshots corresponding to the different strains in the stretch-recovery deformation are prepared in the supporting information (Figure S1). For the SR system (Nc=400), the initial box size *L_z_*(0) is 25.2 and the L_z_ at the maximum strain is 148.6 during the tensile process. For the fixed junction system (Nc=400), the initial box size *L_z_*(0) is 22.5, and the *L_z_* at the maximum strain is 132.9, during the tensile process. Similarly, during the compressive process, *L_z_* at the maximum strain is 12.8 and 11.5 for the SR system (Nc=400) and fixed junction system (Nc=400), respectively.

All our MD simulations are carried out through the large scale atomic/molecular massively parallel simulator (LAMMPS) [45] developed by the Sandia National Laboratories, and some more detailed simulation techniques can be found in our previous work [46,47,48].

## 3. Results and Discussion

### 3.1 Dynamic Properties

Firstly, we study the differences in the dynamics between SR systems and the fixed junction systems. All the systems are simulated in the NPT ensemble for the calculation of all the dynamic properties, including mean-square displacement (MSD) [49,50,51,52], the bond autocorrelation function [53], and the end-to-end correlation function [54,55].

**Figure 2:**
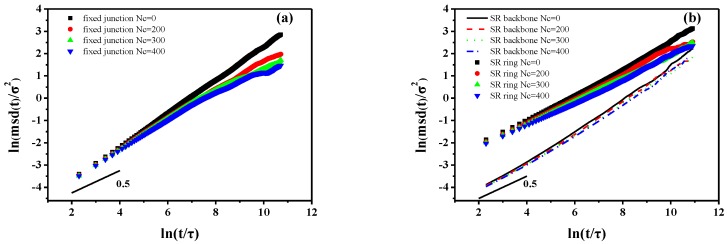
The mean-square displacement (MSD) of (**a**) backbone in fixed junction systems and (**b**) backbone and ring structure in SR systems. The black lines with slope of 0.5 are shown as a guide for the eye.

The MSD curves are always used to demonstrate the translational mobility, listed as follows:

(7) $$msd(t)=\frac{1}{N}\sum_{i=1}^{N}\langle {\left|r_{i}(t+t_{0})-r_{i}(t_{0})\right|}^{2}\rangle$$

in which *N* is the number of backbones or rings. The averaging is performed over all the repeating units (backbone or ring). The MSD is calculated by using 60,000*τ* simulation trajectories with time increments of 10*τ* in each case. As Figure 2 shows, the transitional mobility of backbone in fixed junction systems is higher than that of the SR systems. Notably, the initial values of MSD log-log curve are negative, because we apply the log-log format of the MSD figure. In addition, the curves in Figure 2a,b show an initial slope of ~0.5 that is in agreement with the Rouse model [56,57,58]. After the initial *t*^1/2^ time, there is a distinct decrease trend in the slopes describing backbones in fixed junction systems and rings in SR systems. For the system with relatively higher *Nc*, two subdiffusive regimes for MSD are observed. However, the MSD of backbones in SR shows linear diffusion behavior similar to the case of no cross-linked fixed junction system. Notably, the effect caused by Nc is a little different between the trend of MSD in rings and backbone.

**Figure 3:**
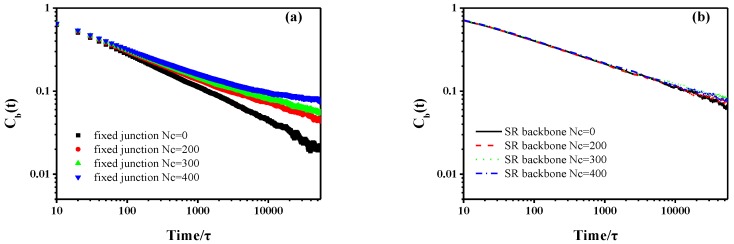
The bond autocorrelation function of (**a**) backbone in fixed junction systems and (**b**) backbone in SR systems.

**Figure 4:**
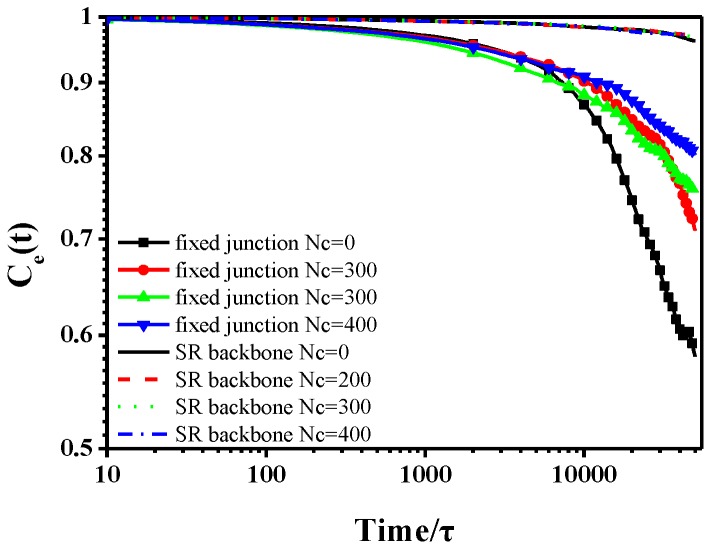
The end-to-end autocorrelation function of backbone in fixed junction systems and SR systems.

The bond autocorrelation function and the end-to-end autocorrelation function are also used to reveal the relaxation of the backbone, by describing the rotational motion in all systems:

(8) $$C(t)=\langle C(t)\cdot C(t=0)\rangle \;(C(t)=C_{b}(t)\;\mathrm{or}\;C_{e}(t))$$

in which $C_{b}(t)$ and $C_{e}(t)$ represent the bond autocorrelation function and the end-to-end autocorrelation function, respectively. Also, C(t) and C(t=0) stand for the unit vector of each bond or end-to-end vector in the backbone at time t and the initial time, t=0. Both are used to characterize the rotational motions of the backbone, as shown in Figure 3 and Figure 4. The time scale is consistent with the MSD curves in Figure 2. Qualitatively, the behavior of SR systems is similar, which also shows similar dynamics to the case of no cross-linked fixed junction systems, according to Figure 3. However, in Figure 3 and Figure 4, the dynamics of SR systems are much slower than those of the cross-linked fixed junction systems.

**Figure 5:**
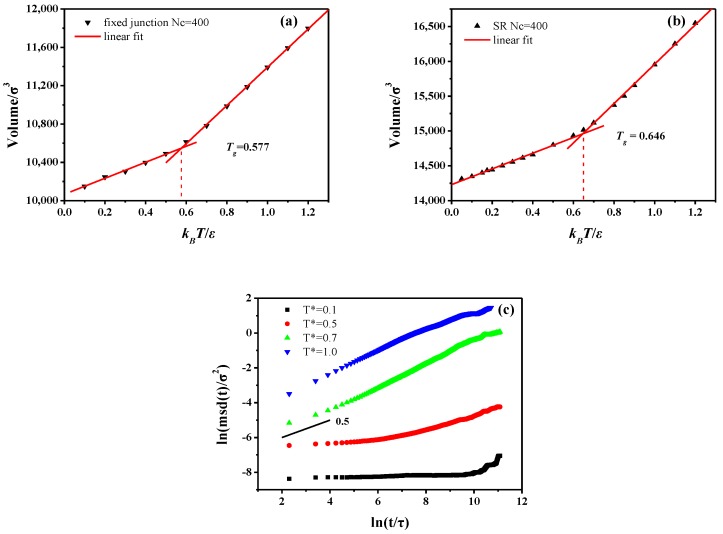
Temperature dependence of the volume figuring out the glass-transition temperature “volumetric $T_{g}$”of the (**a**) fixed junction system when Nc=400 and (**b**) SR system when Nc=400. The red lines represent the linear fitting curve. (**c**) The mean-square displacement (MSD) of backbone in fixed junction systems (Nc=400) at different temperatures. Notably the “dynamic $T_{g}$” of this system is around 0.6.

Additionally, we characterize the glass transition by plotting the change of the specific volume as a function of the temperature. Notably, the translational and rotational dynamics are all examined in the melt state ($T^{*}=1.0$). We compare the fixed junction and the SR systems by fixing the cross-linked bonds Nc=400. The $T_{g}$ of SR system is $T_{g}=0.646$, while it is $T_{g}=0.577$ for the fixed junction system, as shown in Figure 5a,b. Meanwhile, compared to the measure of “volumetric $T_{g}$” above, we plot the MSD curves for the fixed junction system (*Nc* = 400) at various temperatures to show the change of the backbone motion when crossing $T_{g}$. As Figure 5c shows, the MSD curves show completely different diffusive behavior between $T^{*}=0.5$ and $T^{*}=0.7$, which indicates the “dynamic $T_{g}$” is around 0.6 and also in agreement with the $T_{g}$ calculated by the temperature-volume relation. In addition, the differences in the diffusive behavior are attributed to the “cage effect”. At the initial time, the segment is surrounded by other segments, which leads to its extremely slow motion. After a certain time, this segment will have the chance to escape this confined cage, changing to be in the state of fast motion. Obviously, the SR system has a much higher glass transition temperature, indicating relatively slower chain dynamics [59]. The observation is in accordance with the results indicated by the mean-square displacement (MSD), the bond autocorrelation function, and the end-to-end autocorrelation function.

### 3.2. Stretch-Recovery Behavior

**Figure 6:**
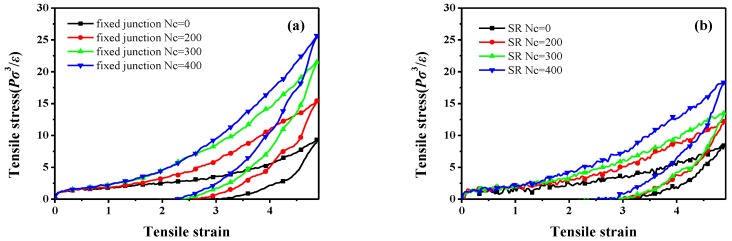
Stretch-recovery curves for different number of cross-linked bonds, and Nc in (**a**) the fixed junction systems and (**b**) SR systems.

Having revealed the effect of the SR junction on the dynamic properties, we now discuss the results for comparison on stretch-recovery behavior. Figure 6 demonstrates the stretch-recovery curves with different Nc.

We examine the stretch-recovery behavior and the bond orientation in the *z* direction. The stress-strain curves are both significantly enhanced with an increase of Nc, as shown in Figure 6. Compared with the stretch-recovery curves of the fixed junction systems (Figure 6a), the SR systems show much lower mechanical properties at the same Nc (Figure 6b), which is in good agreement with the result of Ito [23]. We infer that the decrease of the stress may be caused by the “pulley effect”, the backbone chain sliding through the rings, and the re-distribution of the stress along the backbone.

**Figure 7:**
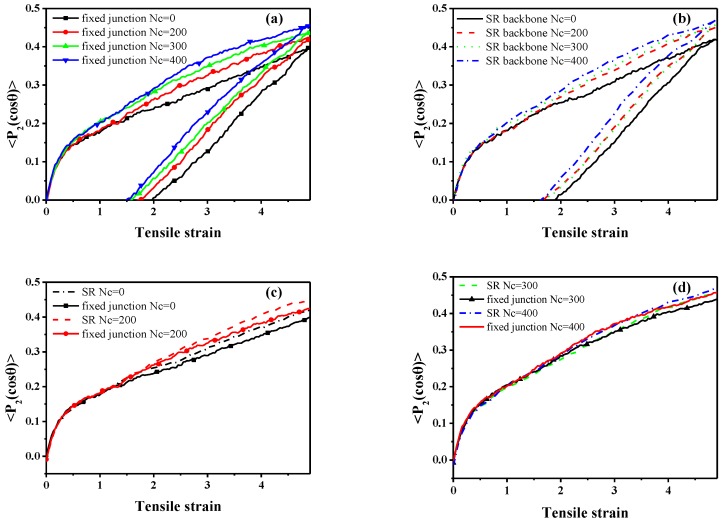
The orientation of the bond, $\langle P_{2}\left(\cos\theta \right)\rangle$, in backbone during the stretch-recovery deformation in the *z* direction for different number of cross-linked bonds; Nc within (**a**) the fixed junction systems and (**b**) SR systems; (**c**) comparison of the $\langle P_{2}\left(\cos\theta \right)\rangle$ of backbone in the different system when Nc=0, 200 during the stretch deformation; (**d**) comparison between the $\langle P_{2}\left(\cos\theta \right)\rangle$ of backbone in the different systems when Nc=300, 400 during the stretch deformation.

Meanwhile, to quantify the mechanical properties, the orientation of the bond in backbone during the stretch-recovery process is shown in Figure 7, which is characterized by the second-order Legendre polynomials $\langle P_{2}(\cos\theta )\rangle$ [60]:

(9) $$\langle P_{2}(\cos\theta )\rangle =(3\langle \cos^{2}\theta \rangle -1)/2$$

in which *θ* denotes the angle between a given bond (adjacent bead in the backbone structure) and the deformation direction *z*. However, in SR systems, the orientation of backbones is markedly enhanced with an increment in Nc, which is identical to fixed junction systems, as Figure 7 shows. According to the comparison of bond orientation with the same Nc shown in Figure 7c,d, the $\langle P_{2}(\cos\theta )\rangle$ in SR system with Nc=0 and Nc=200 is higher than that in fixed junction systems with the same Nc, while the difference becomes smaller when Nc=300 and Nc=400.

According to the theory of Rubinstein [61], a large permanent set usually indicates great chain slippage and leads to the significant hysteresis loss. Here, the “permanent set” is defined as the value of the strain, while the stress decreases to zero during the recovery process. Therefore, the comparison of the permanent set after the stretch-recovery is presented in Table 3, corresponding to the stretch-recovery curves in Figure 6. Remarkably, the values of permanent set in the SR systems are much bigger than those in the fixed junction cases, as shown in Figure 6. According to the orientation of chain backbone along the deformed direction (Figure 7c,d), there is a much bigger bond orientation for the SR systems compared with the fixed junction case. Thus, it is difficult for all the SR systems to recover to their initial state, resulting in a much larger permanent set after the stretch-recovery process.

**Figure 8:**
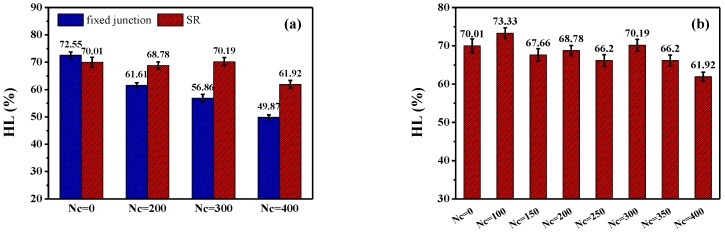
Hysteresis loss (HL) during the stretch-recovery deformation for different numbers of cross-linked bonds; Nc in (**a**) the fixed junction systems and SR systems and (**b**) HL of SR systems with a subdivided range of Nc from 0 to 400.

Meanwhile, in order to quantitatively reveal the effect of the cross-linked ring network on the visco-elasticity and damping property, we compare the hysteresis loss (HL), defined as the ratio of the loss energy to the storage energy within the stretch-recovery cycle in all systems. All the simulated systems are in their visco-elastic state, because the simulated temperature of all systems is above $T_{g}$. All the stretch-recovery cycles in Figure 6a,b are adopted for the comparison of HL, and finally the statistics are shown in Figure 8. Obviously, according to Figure 8, the HL of the fixed junction systems decreases rapidly with the increase of Nc. However, the HL of SR systems is much bigger than that in the fixed junction systems at the same Nc, even though the HL values of the SR systems cannot exhibit a distinct trend with the Nc increasing, as Figure 8a shows. Here, the prominent HL in the SR systems results from the “pulley effect”, the rings’ sliding motion. Therefore, the cross-linked ring network formed in the SR may contribute to a remarkable augmentation in the hysteresis loss during the stretch-recovery process. Furthermore, the detail HL values of the SR systems with Nc=0, 100, 150, 200, 250, 300, 350, and 400 are calculated and presented in Figure 8b. During the stretch-recovery simulation, the SR systems show lower reinforcing mechanical properties but process much more hysteresis loss compared with the fixed junction systems.

To better analyze the phenomenon above, the averaged energy of non-bonding interaction ($E_{\mathrm{non}-\mathrm{bonding}}$), bond stretching energy ($E_{\mathrm{bond}}$), and their variations of the whole stretching process ($\Delta E_{\mathrm{non}-\mathrm{bonding}}$, $\Delta E_{\mathrm{bond}}$) are calculated and shown in supporting information (Figure S2). To sum up, it is obvious that all the $\Delta E_{\mathrm{bond}}$ values in SR systems are lower than those of fixed junction systems, which could rationalize the decrease of the mechanical property of the SR systems results from the much weaker bond stretching compared with the fixed junction systems. Besides, greater $\Delta E_{\mathrm{non}-\mathrm{bonding}}$ also accounts for the more prominent orientation of chain backbones along the *z* direction in SR systems.

**Figure 9:**
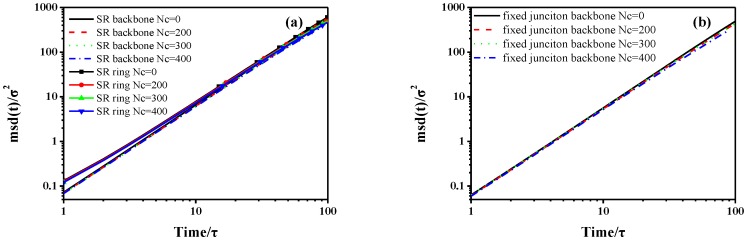
During the stretch process, the mean-square displacement (MSD) of (**a**) backbone and ring in SR systems; (**b**) backbone in the fixed junction systems with different number of cross-linked bonds, Nc. The curves are derived from statistical averages and each curve consists of 100 data points. The Figure 9 is the log-log version of the Figure S4.

**Figure 10:**
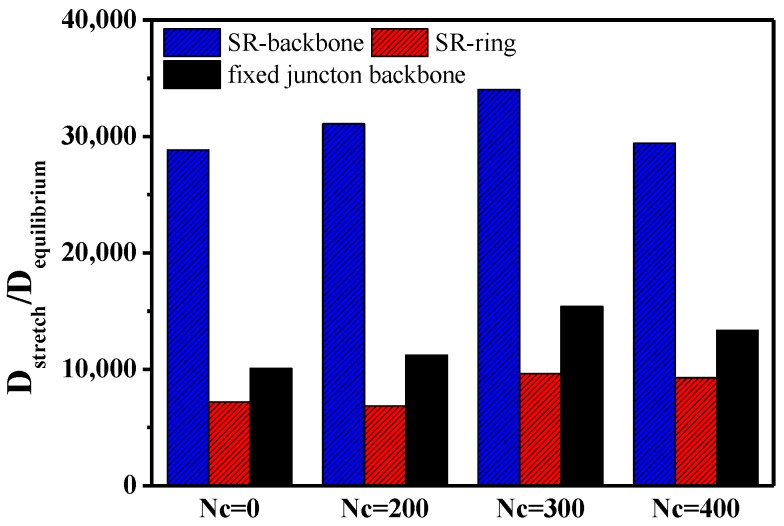
The comparison of $D_{\mathrm{stretch}}/D_{\mathrm{equilibrium}}$ between different motional units in SR systems and fixed junction systems. The diffusion coefficients are derived from linear fitting on the MSD curves under stretch and equilibrium without deformation.

Apart from the evolution of interaction during the stretch deformation, we want to probe the “pulley effect” attributed to the relative motion in SR systems. Based on the requirement, we plot the curves describing the mean-square displacement (MSD) of backbone, ring in SR systems, and the backbone in the fixed junction systems during the stretching process, compared with that during the equilibrium process in Section 3.1. As Figure S4a shows, the MSD curve of ring structure is still above that of the backbone structure at the same Nc in SR systems. Also, the MSD in ring structure decreases with the increment of Nc, from 0 to 400, which is identical to the result in the dynamic properties mentioned above. According to Figure S4b, there is also a general decrease trend in the MSD, with Nc increasing in the fixed junction systems. Considering the different motional units, we cannot directly compare the MSD curves and the diffusion coefficients to analyze the motion, because the size of ring is much smaller than the size of backbone as the model we set. Here, we define a ratio, $D_{\mathrm{stretch}}/D_{\mathrm{equilibrium}}$, to describe the difference between mobility in the equilibrium and stretch process, in which $D_{\mathrm{stretch}}$ stands for the diffusion coefficients under external stress during the stretch process. Besides, the $D_{\mathrm{equilibrium}}$, which is calculated from Figure 2, stands for the dynamics in the equilibrium state without deformation. By that mean, the ratio excludes the effect of the dynamics and illustrates the segment motion under internal stress. The diffusion coefficients (self-diffusion coefficients), *D*, of the chain determined from the Stokes-Einstein equation [49,51]:

(10) $$D=\frac{1}{6}\underset{t\to \infty }{\lim}\frac{d}{dt}\langle {\left[\vec{r}(t)-\vec{r}(0)\right]}^{2}\rangle =\frac{1}{6}\underset{t\to \infty }{\lim}\frac{d\Delta r^{2}}{dt}$$

in which the quantity in 〈…〉 is the ensemble-averaged mean square displacement (MSD) of the center-of-mass of the chain. The diffusion coefficients can be calculated from the MSD curves as 1/6 of the slope form Figure 9 [62]. In addition, all diffusion coefficients are calculated through the linear fitting of the MSD curves. We merely mean to characterize the motion of the repeating units during the equilibrium and deformation, though the “diffusion coefficient” is usually used for equilibrium. The ratio $D_{\mathrm{stretch}}/D_{\mathrm{equilibrium}}$ of different motional units is presented in Figure 10.

The ratios, $D_{\mathrm{stretch}}/D_{\mathrm{equilibrium}}$, for different motion units are greater than 1.0, which means that the segment motion is enhanced by external force during the stretch. Comparing the ratios of backbone in SR systems and fixed junction systems, we found that value of the ratio describing backbone in SR systems is much bigger than that in the fixed junction system. Namely, the enhanced diffusion coefficients of backbone in SR systems attributed to the stretching are much greater than those in the fixed junction systems. Similarly, the motion of the rings is also enhanced, but the variation of the enhancement is not as great as that of backbone in the same SR systems. We infer that the cross-linking between rings may affect the mobility of the rings more than with the backbone. All the facts above indicate that the relative motion attributes to the “pulley effect” between rings, and the mobility of SR systems is significantly promoted during the stretch deformation.

**Figure 11:**
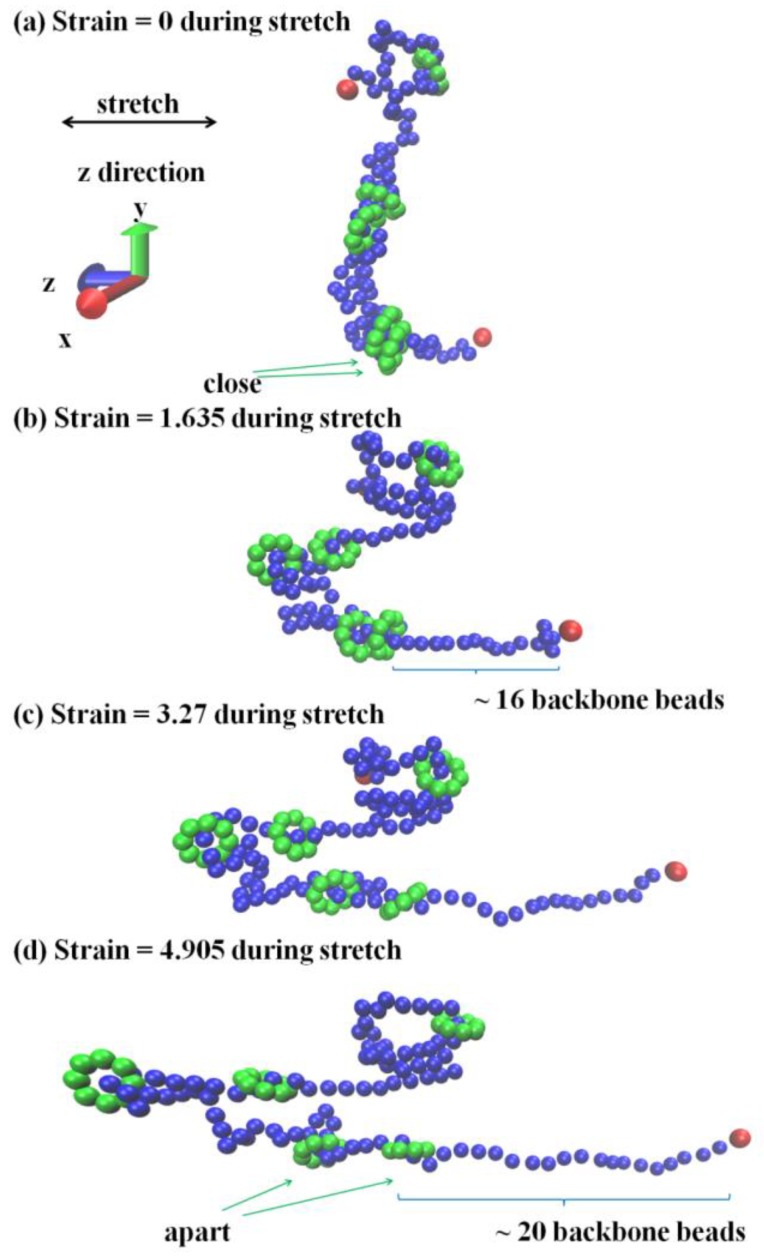
Snapshots of single SR structure corresponding to the different strains in the stretch deformation, monitoring the trajectories and chain sliding.

In fact, a much more apparent characterization on the chain sliding will strongly validate our conclusion. Therefore, a visualized snapshot (by software VMD) has been displayed, which merely but simply shows the chain sliding in the SR system. In Figure 11, one single SR structure from the SR system (Nc=400) is picked to show the chain sliding under loading. Notably, the cross-links are not shown in this figure. At the initial state (Figure 11a), two ring structures (bottom) are close to each other; however, they become separated when the strain is equal to 4.905. Meanwhile, the backbone orientates along the stretching direction, which clearly validates the chain sliding.

To sum up, compared with the fixed junction systems, the SR systems exhibit more complicated properties during the stretch-recovery process, showing lower mechanical property but higher orientation of the backbone along the stretching direction. The chain sliding in SR systems is validated by the relative diffusion coefficients and visualized snapshots.

### 3.3. Compression-Recovery Behavior

**Figure 12:**
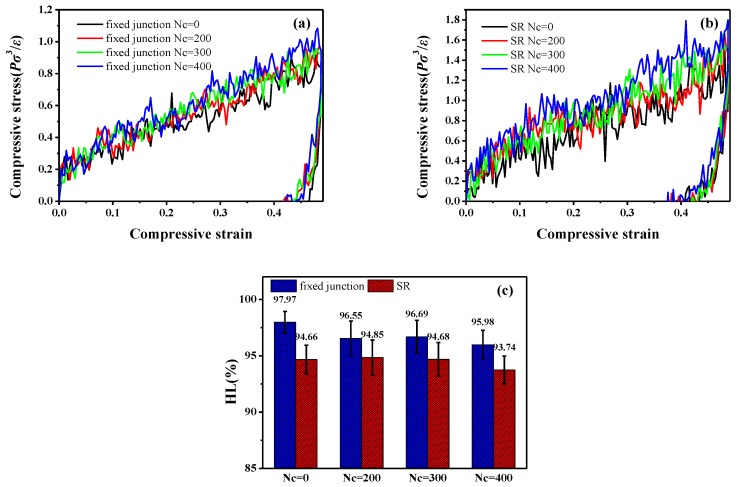
Compression-recovery curves for different number of cross-linked bonds; Nc in (**a**) the fixed junction systems and (**b**) SR systems, (**c**) hysteresis loss (HL) during the compression-recovery process for different number of cross-linked bonds, and Nc in the fixed junction systems and SR systems.

To further explore the effect of the cross-linked ring network on the visco-elasticity, we also examine the compression-recovery conformation. According to the results of experiment in previous report [24], the slide-ring gels show different mechanical properties in the tensile and compressive deformation. In Figure 12a,b, the compression-recovery curves in the fixed junction systems and SR systems are presented. Figure 12a exhibits the compression-recovery curves in the fixed junction systems. With the increment of Nc, the compression stress increases slightly with minor difference, and the maximum of the stress is around $1.0P\sigma^{3}/\varepsilon$. As for the curves in Figure 12b, the SR systems show much bigger difference with the increase of Nc in comparison to the Figure 12a, and the maximum of the stress is around $2.0P\sigma^{3}/\varepsilon$. It is distinct that the mechanical property in SR systems is better than that in the fixed junction during the compression deformation, which is also confirmed in the experiment by Kato et al. [24]. Similarly, the Hysteresis loss (HL) during the compression-recovery process is calculated in Figure 12c. During the whole deformation, HL values are all above 90%, while the HL in the SR systems is slightly smaller than that in the fixed junction systems. The interesting point is that the mechanical property observed in the stretch-recovery process is different from that in the compression-recovery deformation.

**Figure 13:**
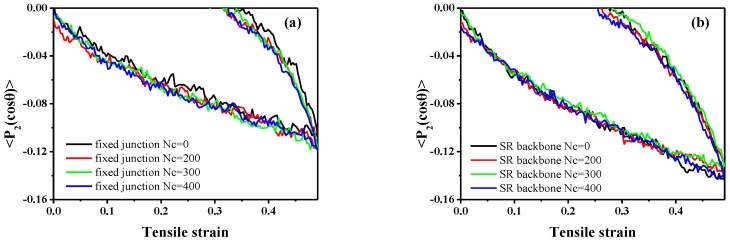
The orientation of the bond, $\langle P_{2}\left(\cos\theta \right)\rangle$, in backbone during the compression-recovery deformation in the *z* direction for different number of cross-linked bonds; Nc within (**a**) the fixed junction systems and (**b**) SR systems.

Furthermore, we also calculate the orientation of the bond in backbone, $\langle P_{2}(\cos\theta )\rangle$, in Figure 13. According to Figure 13a,b, $\langle P_{2}(\cos\theta )\rangle$ decreases from 0 to the negative values with the increase of the compressive strain, so the systems tend to become oriented perpendicularly to the *z* direction, or parallel to the *xoy* plane. Also, the values of $\langle P_{2}(\cos\theta )\rangle$ in the SR systems (Figure 13b) are lower than those in fixed junction systems (Figure 13a), indicating a greater orientation of the bond along the *x* or *y* direction (perpendicular to the compression stain direction, *z*). However, the stress of the SR systems during the compression-recovery process is much bigger than that of the fixed junction systems. According to the research by Kato, the so-called “strain hardening” behavior under compression is also reported, and the loss of the entropy within rings threading on the backbone along the compression direction is responsible for the “strain hardening”. The reinforcement by aggregation of rings finally accounts for the phenomenon [24,44].

**Figure 14:**
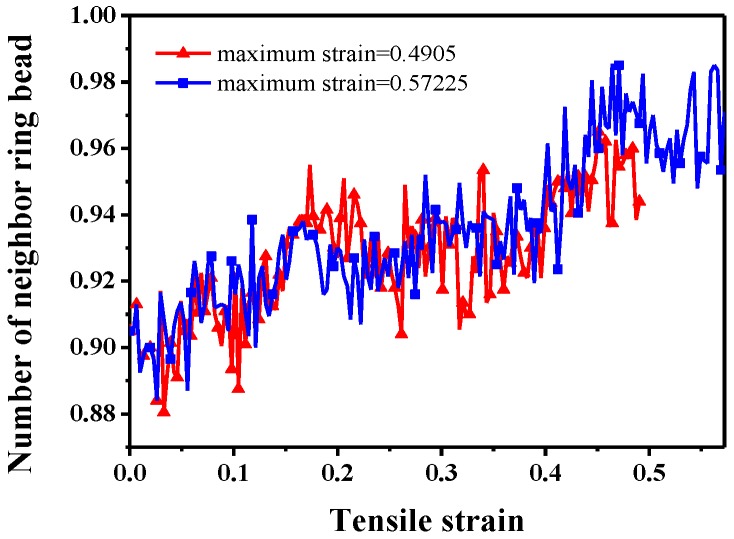
Average number of neighbor ring beads (not including the bead on the same ring) as a function of the strain during the compressive deformation. Two independent compressive deformations are involved in the figure, and the maximum strain is equal to 0.4905 and 0.57225.

Therefore, we characterize the ring aggregation by introducing the “number of neighbor beads” to the compressive process, as we did in the study of characterizing the dispersion state of nanoparticles before [63,64]. To quantitatively characterize the ring aggregation state as a function of the compressive strain, the average number of ring beads on neighbor rings around each bead on ring is calculated within a distance of *L* = 1.12*σ* (cutoff), as Figure 14 shows. The number of neighbor beads validates a gradual aggregation state of rings, though many fluctuations exist.

Meanwhile, the averaged energy of non-bonding interaction, $E_{\mathrm{non}-\mathrm{bonding}}$, and bond stretching energy, $E_{\mathrm{bond}}$, during the compressing deformation are presented in Figure S3. The $\Delta E_{\mathrm{non}-\mathrm{bonding}}$ in SR systems during the whole compression process is greater than that of the fixed junction systems, which supports the result in the orientation of bond for the chain backbone.

**Figure 15:**
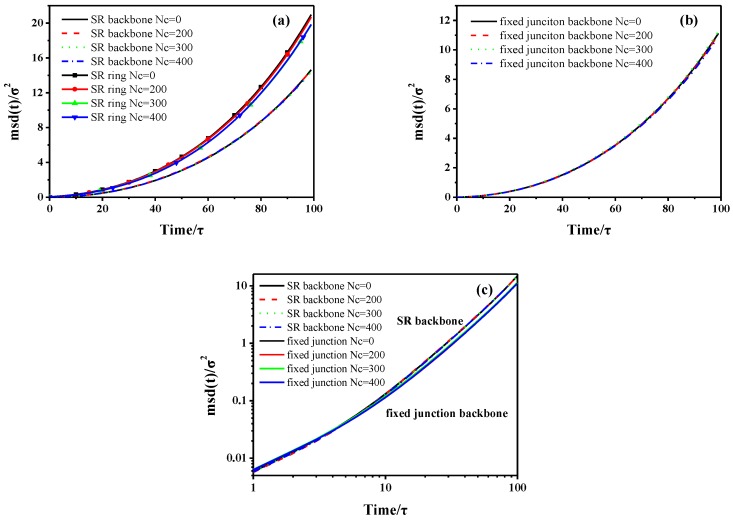
The mean-square displacement (MSD) of (**a**) backbone and ring in SR systems, (**b**) backbone in the fixed junction systems with different number of cross-linked bonds, and Nc during the compressive process. (**c**) The comparison of raw MSD curves of backbone in SR systems and fixed junction systems. Notice that the curves with different *Nc* are aligned to each other. Those curves are derived from statistical averages, and each curve consists of 100 data points.

Similarly, we further demonstrate the “pulley effect” attributed to relative motion in the SR systems during the compression deformation. Then, we plot the curves describing the MSD of different units during the compression deformation, as the Figure 15 shows. Meanwhile, we compare those data with the dynamic properties in the equilibrium process (Section 3.1). In Figure 15a, although the curves of ring are above the backbone and exhibit much greater diffusion coefficients, we cannot compare them directly to the different units of motion. Besides, the MSD of SR systems with different Nc is similar, which also assumes that the cross-linking density shows less effect on the diffusion of stress-strain behavior. Meanwhile, the MSD curves describing the fixed junction systems are in superposition with each other, as presented in Figure 15b.

To probe the “pulley effect” attributed to the relative motion under compression, we intend to apply the same method to compute the relative diffusion during the compressive process; however, the fitting results of the MSD curves in Figure 15a,b were not good enough. Therefore, we compared the raw MSD curves during the compression process. As Figure 15c shows, with the comparison of raw MSD between the backbone in SR systems and fixed junction systems, we can also attribute the much higher translational mobility of SR systems to the chain sliding. This result also accounts for the fact that the orientation of backbone enhanced perpendicularly to the compression direction.

## 4. Conclusions

In this work, the slide-ring (SR) and the fixed junction systems are explored via coarse-grained molecular dynamics simulation. Firstly, we investigate the effect of the cross-linking density on the dynamics such as the mean-square displacement (MSD) and the bond and end-to-end autocorrelation function, concluding that the mobility of SR system is much slower than that of the fixed junction system at the same cross-linking density. The glass transition temperature for the slide-ring system is lower than that of the fixed junction system at the same cross-linking density such as Nc=400, which is accordance with the analysis of the dynamics. After that, by examining the stretch-recovery behavior of SR and fixed junction systems for different cross-link densities, we observe that the chain orientation of the SR systems is slightly higher than that of the fixed junction systems, attributed to the much greater chain sliding, which, however, worsens the tensile stress-strain behavior. Therefore, we infer that the cross-linked SR network could re-distribute the external force and show much smaller tensile stress compared with the fixed junction systems, which is attributed to the “pulley effect”. In addition, larger permanent set and higher hysteresis loss also verify the chain sliding in SR systems. Furthermore, we define the ratio of the diffusion coefficient of polymer chains and rings $D_{\mathrm{stretch}}/D_{\mathrm{equilibrium}}$ between the tensile state and the equilibrium state and then obtain the sliding motion confirmed again. Meanwhile, during the compression-recovery process, a much higher chain orientation is observed in the SR systems compared with the fixed junction systems, which is attributed to the sliding motion. Besides, in contrast to the fixed junction, the increased compressive stress in SR systems results from the aggregation of the rigid rings. To sum up, the slide-ring junction exhibits the unique mechanical behavior compared with the fixed junction, especially the capability to sustain external force and possess high hysteresis. Our work provides molecular insights into the slide-ring junction structure and makes it possible to further guide the design and preparation of the slide-ring material with high damping and good mechanical properties.

## Figures and Tables

**Table 1:** Potential parameters of the non-bonding interaction.

Non-Bonding Pair		$\varepsilon_{\mathrm{ij}}(\varepsilon )$	σ(σ)	$r_{\mathrm{EV}}(\sigma )$	$r_{\mathrm{cutoff}}(\sigma )$
backbone	backbone	1.0	1.0	0	2.5
backbone	end	5.0	1.0	0.5	2.24
backbone	ring	1.0	1.0	0	2.24
end	end	1.0	1.0	1.0	1.12
end	ring	1.0	1.0	0.5	1.12
ring	ring	1.0	1.0	0	1.12

**Table 2:** Parameters for the bond stretching interaction.

Bond Stretching	$k(\varepsilon \cdot \sigma^{-2})$	$r_{0}(\sigma )$
backbone–backbone	100	1.0
cross-linking bond inter-backbone	100	1.0
backbone–end	180	1.5
cross-linking bond inter-ring	100	1.0

**Table 3:** Permanent set in all systems after the stretch-recovery deformation.

Nc	Permanent Set after Stretch-Recovery Deformation	
	Fixed Junction	SR
0	3.10	3.01
200	2.61	3.01
300	2.42	2.97
400	2.29	2.65

## Supplementary Materials

The following are available online at http://www.mdpi.com/2073-4360/10/9/964/s1.

## Nomenclature

length parameter	σ
Well depth in LJ interaction	ε
reduced time	$t^{*}=t/\tau ;\;\tau =\sigma \sqrt{m/\varepsilon }$
reduced temperature	$T^{*}=\left(k_{B}T/\varepsilon \right)$
reduced density	$\rho^{*}=\rho \sigma^{3}$
reduced pressure	$P^{*}=\left(P\sigma^{3}/\varepsilon \right)$
reduced energy	$E^{*}=E/\varepsilon$
reduced volume	reduced volume
