# Supplementary material for: Designing the Slide-Ring Polymer Network with both Good Mechanical and Damping Properties via Molecular Dynamics Simulation
Source: Polymers (Basel). 2018 Sep 1;10(9):964. doi: 10.3390/polym10090964 (PMC6403985; doi:10.3390/polym10090964)
Supplement: Supplementary file 1 [file polymers-10-00964-s001.pdf]

Supporting information

Designing the slide-ring polymer network with both good mechanical and damping properties via molecular dynamics simulation

Zhiyu Zhang[1], Guanyi Hou[1], Jianxiang Shen[2], Jun Liu[1,3,4,5*],Yangyang Gao[1], Xiuying Zhao[1], Liqun Zhang[1,3,4,5,6*]

[1]Key Laboratory of Beijing City on Preparation and Processing of Novel Polymer Materials, Beijing University of Chemical Technology, People's Republic of China

[2]Department of Polymer Science and Engineering, Jiaxing University, Jiaxing 314001, People's Republic of China

[3]Beijing Engineering Research Center of Advanced Elastomers, Beijing University of Chemical Technology, People's Republic of China

[4]Engineering Research Center of Elastomer Materials on Energy Conservation and Resources, Beijing University of Chemical Technology, People's Republic of China

[5]Beijing Advanced Innovation Center for Soft Matter Science and Engineering, Beijing University of Chemical Technology, 100029 Beijing, People's Republic of China

[6]State Key Laboratory of Organic-Inorganic Composites, Beijing University of Chemical Technology, 100029 Beijing, People's Republic of China

Supporting information

Method to building the systems

Figure S1-S6

*Corresponding author: liujun@mail.buct.edu.cn or zhanglq@mail.buct.edu.cn

Method to building the systems

The systems are constructed by replicating one linear backbone molecule (for fixed junction systems) or one SR structure (for SR structure) shown in the Fig. 1. Notably, the SR structure is constructed by artificially assembling the rings and linear backbone. Using terminal beads to guarantee the integrity of the SR structure is essential for maintaining the slide-ring structure.

After a long period of equilibrium, the cross-links in the structure were also introduced by the C++ programming and simplified with a relatively random process. Here we define that the cross-linking occurs between beads on different chain(or different rings) when two beads are close enough to each other(or within a certain distance). So we can regulate the number of cross-links and get the network well-distributed.

Figure S1

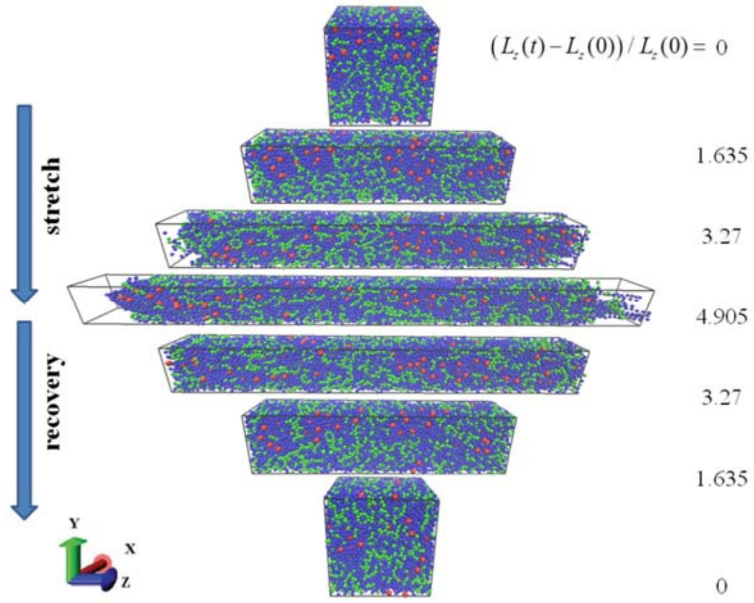

Fig.S1 Snapshots corresponding to the different strains during the stretch-recovery deformation.

Figure S2

All $E_{\text{non-bonding}}$ during the stretch process is negative while the E_{bond} is positive via the calculation of the Eq. (2) and Eq. (3), respectively. According to Fig. S2 (a), the decreasing trend in absolute values of the non-bonding interaction with the increase of the strain is consistent with the increasing trend of the stress-strain curves. During the stretching process, all absolute values of $E_{\text{non-bonding}}$ during the stretch deformation decrease with Nc in Fig. S2 (a). In addition, according to Fig. S2 (b), all $\Delta E_{\text{non-bonding}}$ values with different Nc in SR systems are greater than those of the fixed junction cases.

Meanwhile, the bond stretching energy, E_{bond} is enhanced with the increase of the tensile strain in Fig. S2 (c). We infer that the stretch stress is contributed much from the bond stretching. Notably, the change of the E_{bond}, ΔE_{bond} within the whole stretching process are presented in Fig. S2 (d). Firstly, ΔE_{bond} in the two kinds of systems is enhanced with Nc ranging from 0 to 400, attributed to the fact that the cross-linking density contributes a lot to the bond stretching energy. To sum up, it is obvious that all the ΔE_{bond} values in SR systems are lower than those of fixed junction systems, which could rationalize the decrease of the mechanical property of the SR systems because of the much weaker bond stretching compared to the fixed junction systems. Besides, a greater $\Delta E_{\text{non-bonding}}$ can also account for the more prominent orientation of chain backbones in the SR systems.

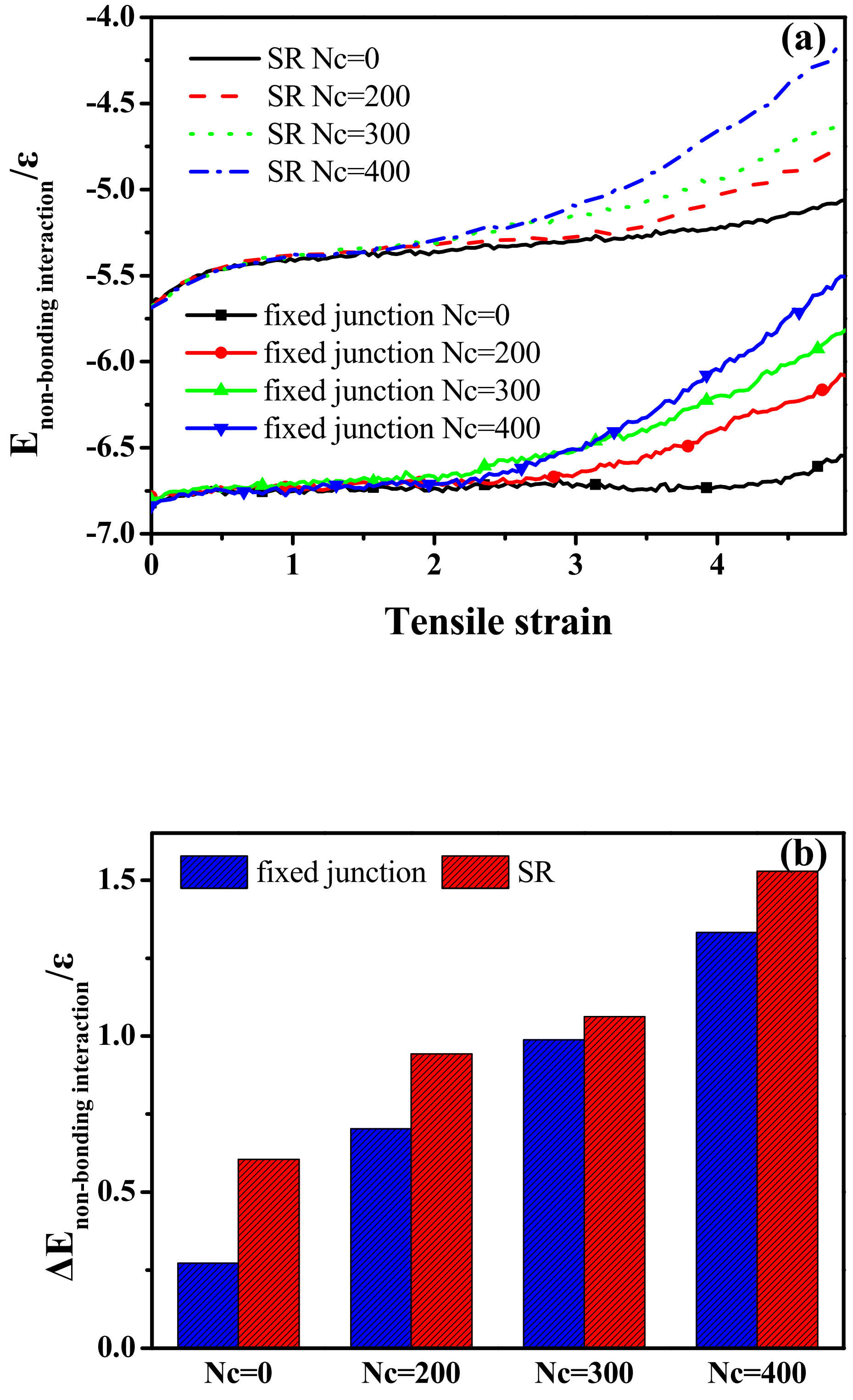

(a)
SR Nc=0
SR Nc=200
SR Nc=300
SR Nc=400
fixed junction Nc=0
fixed junction Nc=200
fixed junction Nc=300
fixed junction Nc=400
E non-bonding interaction/ε
Tensile strain
-4.0
-4.5
-5.0
-5.5
-6.0
-6.5
-7.0
0
1
2
3
4
(b)
fixed junction
SR
ΔE non-bonding interaction/ε
1.5
1.0
0.5
0.0
Nc=0
Nc=200
Nc=300
Nc=400

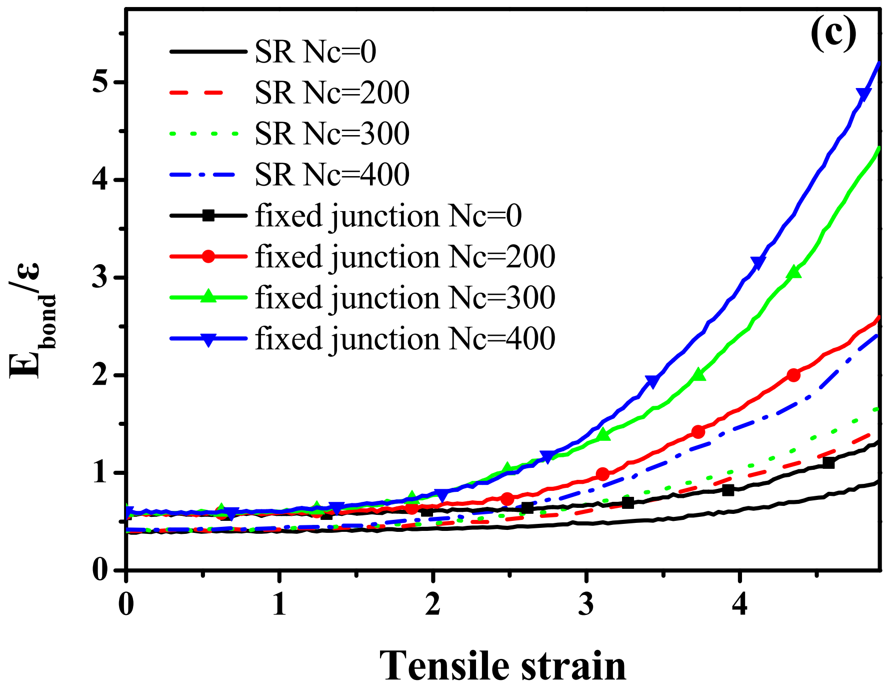

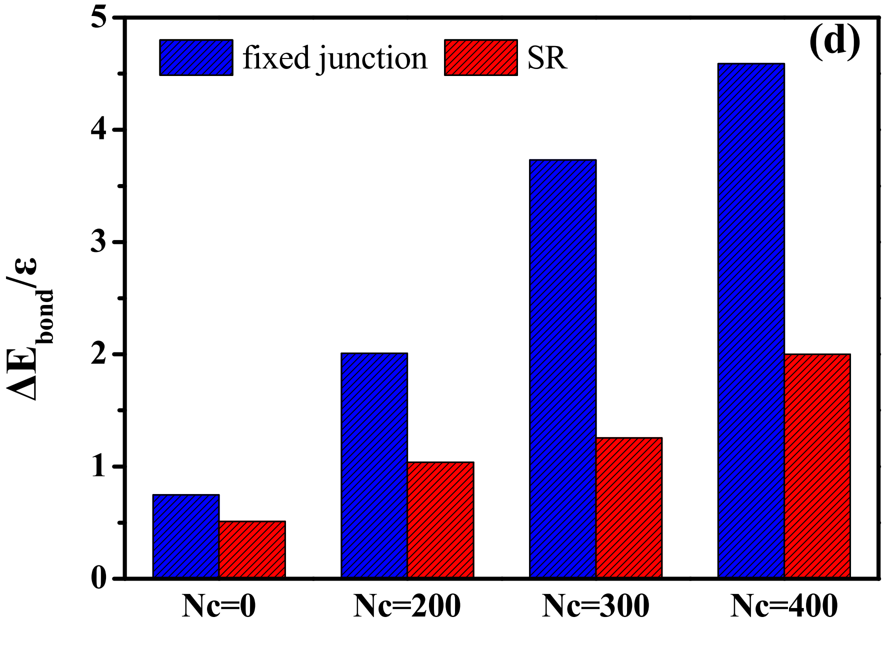

Fig. S2 Comparison of the (a) non-bonding interaction; (b) $\Delta E_{\text{non-bonding interaction}}$; (c) bond stretching energy; (d) ΔE_{bond} with different Nc during the stretch deformation. The $\Delta E_{\text{non-bonding interaction}}$ and ΔE_{bond} stand for the extent of the non-bonding interaction and bond stretching energy within the whole stretch process, respectively.

Figure S3

The averaged energy of non-bonding interaction, $E_{\text{non-bonding}}$ and bond stretching energy, E_{bond} during the compression deformation are presented in Fig. S3. Fig. S3 (a) indicates that the $E_{\text{non-bonding}}$ in SR systems decrease slightly with the strain increasing, while the fixed junction systems show a plateau but exhibit a larger absolute value. Namely, the $\Delta E_{\text{non-bonding}}$ during the whole compression in SR systems is greater than that of the fixed junction systems, which is identical to the result in the orientation of bond for the chain backbone. The $E_{\text{non-bonding}}$ with different Nc during the compression is approximately the same. We infer that the cross-linking density contributes less to the non-bonding interaction during the compression. In addition, Fig. S3 (b) shows a noticeable difference of E_{bond} with Nc increasing.

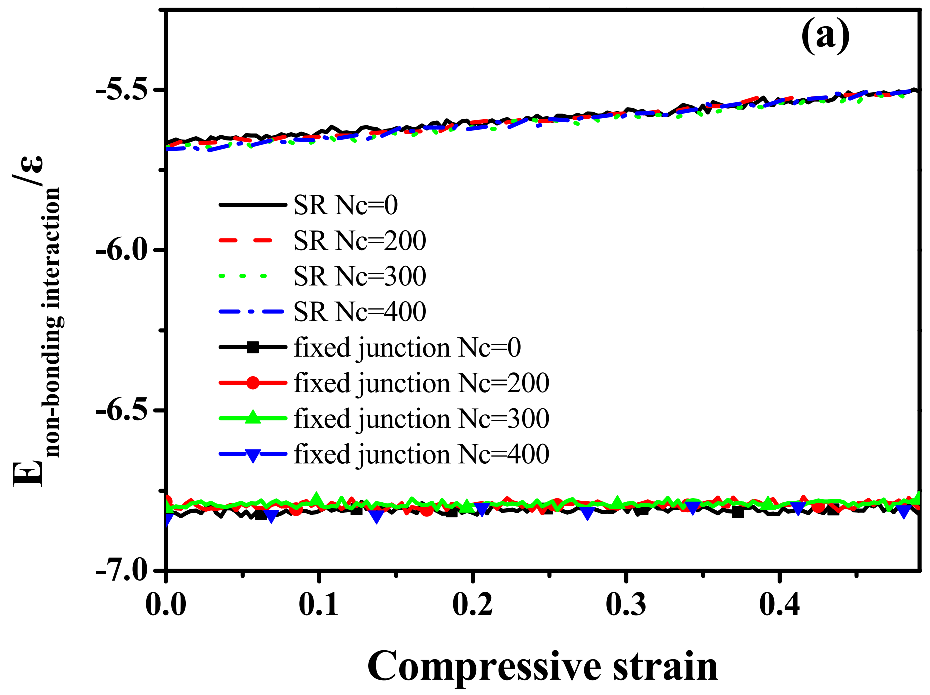

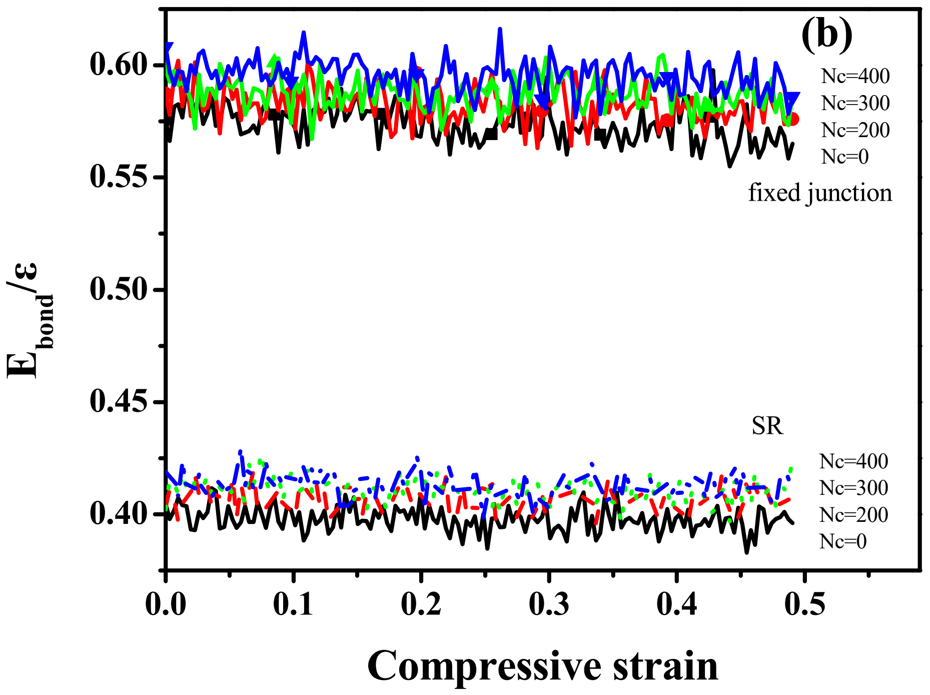

Fig. S3 Comparison of the (a) non-bonding interaction and (b) bond stretching energy for different number of cross-linked bonds, *Nc* during the compression deformation.

Figure S4

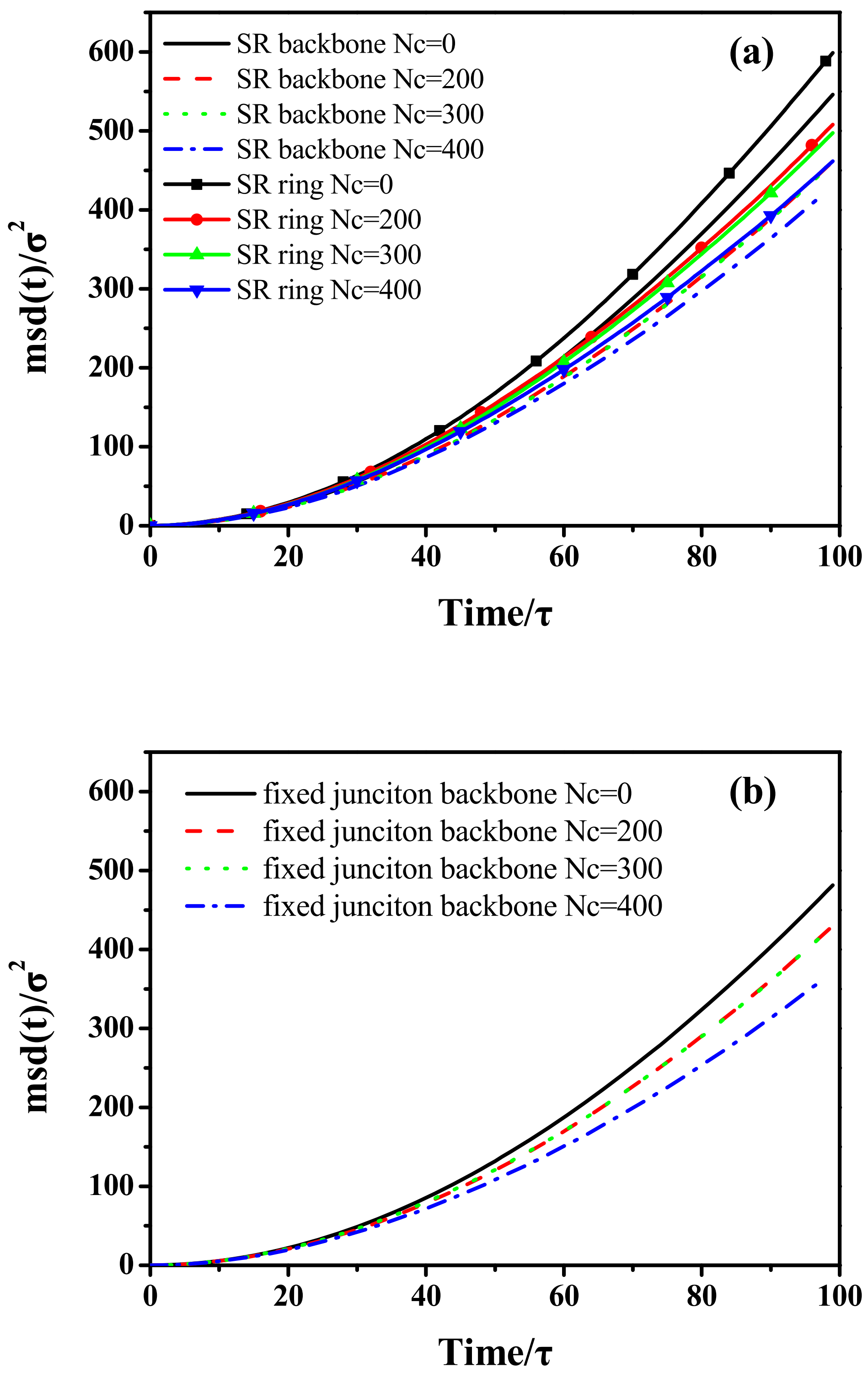

Fig. S4 During the stretch process, the mean-square displacement (MSD) of (a) backbone and ring in SR systems; (b) backbone in the fixed junction systems with different number of cross-linked bonds, Nc.

Figure S5

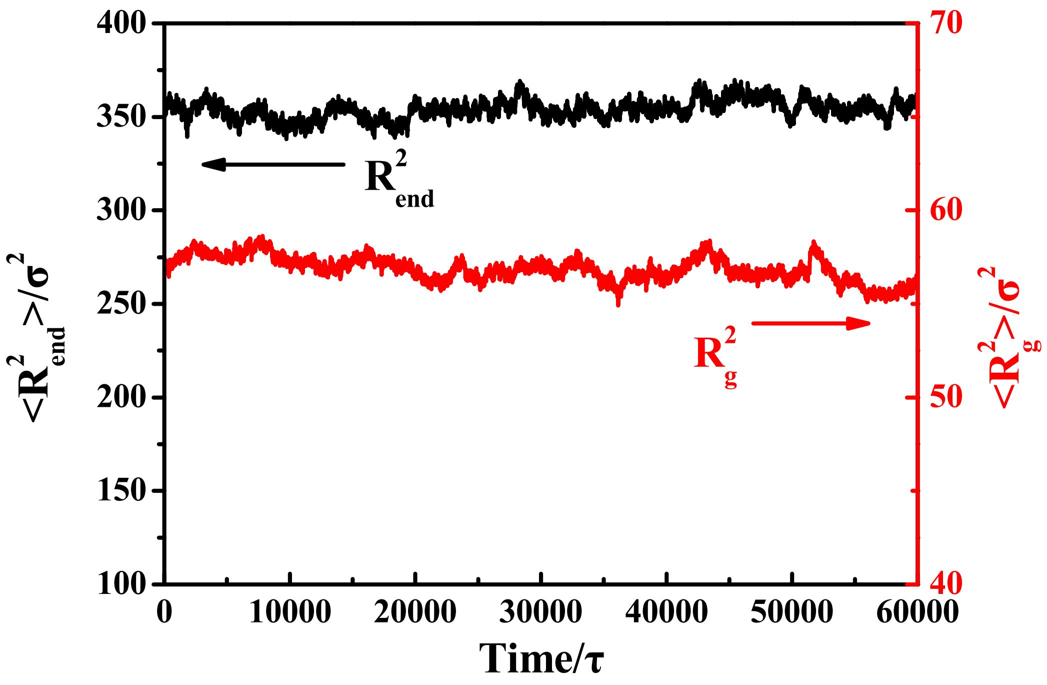

Fig. S5 The mean square end-to-end distance R_{end}^2 and the radius of gyration R_g^2 of the SR systems ($Nc = 400$) during the 6×10^7 timesteps after the initial equilibration.

Figure S6

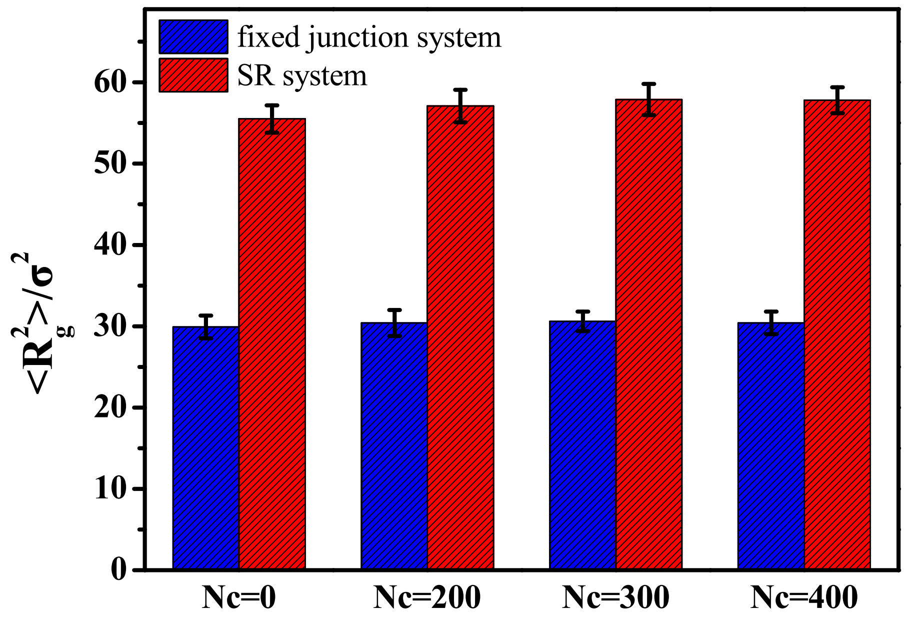

Fig. S6 The mean square radius of gyration R_g^2 of the backbone in all systems during the 6×10^7 timesteps after the initial equilibration (NPT ensemble with $T^*=1.0$ for 6×10^7 timesteps)
